# Effect of mobile application types on stroke rehabilitation: a systematic review

**DOI:** 10.1186/s12984-023-01124-9

**Published:** 2023-01-24

**Authors:** Stephen G. Szeto, Hoyee Wan, Mohammad Alavinia, Sean Dukelow, Heather MacNeill

**Affiliations:** 1 Division of Physical Medicine & Rehabilitation, Department of Medicine, University of Toronto, Toronto, Canada; 2 Division of Plastic, Reconstructive, and Aesthetic Surgery, University of Toronto, Toronto, Canada; 3 Division of Physical Medicine & Rehabilitation, Department of Clinical Neurosciences, Cumming School of Medicine, University of Calgary, Calgary, Canada; 4 Physical Medicine & Rehabilitation, Hennick Bridgepoint Hospital, Sinai Health System, Toronto, Canada; 5 UHN Toronto Rehab Institute, 550 University Avenue, Toronto, ON M5G 2A2 Canada

**Keywords:** Mobile application, Stroke rehabilitation, Systematic review

## Abstract

**Background:**

Stroke is a significant contributor of worldwide disability and morbidity with substantial economic consequences. Rehabilitation is a vital component of stroke recovery, but inpatient stroke rehabilitation programs can struggle to meet the recommended hours of therapy per day outlined by the Canadian Stroke Best Practices and American Heart Association. Mobile applications (apps) are an emerging technology which may help bridge this deficit, however this area is understudied. The purpose of this study is to review the effect of mobile apps for stroke rehabilitation on stroke impairments and functional outcomes. Specifically, this paper will delve into the impact of varying mobile app types on stroke rehabilitation.

**Methods:**

This systematic review included 29 studies: 11 randomized control trials and 18 quasi-experimental studies. Data extrapolation mapped 5 mobile app types (therapy apps, education apps, rehab videos, reminders, and a combination of rehab videos with reminders) to stroke deficits (motor paresis, aphasia, neglect), adherence to exercise, activities of daily living (ADLs), quality of life, secondary stroke prevention, and depression and anxiety.

**Results:**

There were multiple studies supporting the use of therapy apps for motor paresis or aphasia, rehab videos for exercise adherence, and reminders for exercise adherence. For permutations involving other app types with stroke deficits or functional outcomes (adherence to exercise, ADLs, quality of life, secondary stroke prevention, depression and anxiety), the results were either non-significant or limited by a paucity of studies.

**Conclusion:**

Mobile apps demonstrate potential to assist with stroke recovery and augment face to face rehabilitation, however, development of a mobile app should be carefully planned when targeting specific stroke deficits or functional outcomes. This study found that mobile app types which mimicked principles of effective face-to-face therapy (massed practice, task-specific practice, goal-oriented practice, multisensory stimulation, rhythmic cueing, feedback, social interaction, and constraint-induced therapy) and education (interactivity, feedback, repetition, practice exercises, social learning) had the greatest benefits.

*Protocol registration* PROPSERO (ID CRD42021186534). Registered 21 February 2021

**Supplementary Information:**

The online version contains supplementary material available at 10.1186/s12984-023-01124-9.

## Background

Stroke continues to be a leading cause of worldwide disability and morbidity amongst all cardiovascular diseases [1]. From 1990 to 2019, strokes had a global rise in prevalence reaching 101 million people and causing a loss of 143 million disability-adjusted life years [1] at a cost of billions of dollars per year to North American economies [2, 3]. Stroke rehabilitation includes an organized interdisciplinary team approach to stroke specific therapy, and is a critical component of recovery and successful re-integration into society [4]. Compared with other acute stroke interventions, stroke rehabilitation has been found to be as effective or superior to thrombolysis or aspirin [5, 6]. On a per dollar value, the clinical benefits of stroke rehabilitation have been shown to outweigh its costs significantly [7].

Current Canadian Stroke Best Practices and American Heart Association guidelines state that inpatient stroke rehabilitation should provide task-specific therapy (defined as physiotherapy, occupational therapy, and speech and language therapy), for at least 3 h per day of 5 days per week [8, 9]. Evidence supports that more therapy results in improved outcomes [10]. Unfortunately, many institutions struggle to provide this rehabilitation intensity. A 2018 Canadian study found inpatients in a stroke rehabilitation participated in 8.5 h per week of therapy, much below the guideline recommendations of 15 h per week [11]. Outside of therapy, stroke rehabilitation inpatients spend most of their days sedentary [11–13]. There are numerous barriers for meeting current stroke rehabilitation guidelines, including insufficient staff, timing mismatch with other patient activities such as investigations for stroke work-up (CTs, echocardiograms, Holter monitors), and a rise in the number of patients requiring stroke rehabilitation [14, 15].

Mobile applications (apps) for stroke rehabilitation have become an emerging area of interest because of their mobility, multi-functional capabilities such as reminders and videos, and their ability to give patients autonomy over therapy [16–18]. A 2018 systematic review defined several mobile apps for stroke rehabilitation with the potential to be clinically effective [19]. For example, there are mobile apps designed as games to improve finger dexterity, programs to increase exercise adherence, home exercise programs for upper limb rehabilitation, and mirror therapy for facial paresis [16–18, 20]. Mobile apps can target different aspects of stroke rehabilitation. The purpose of this study is to review the effect of mobile apps for stroke rehabilitation on stroke-related impairments and functional outcomes. Specifically, this paper will delve into the effect of varying mobile app types on stroke rehabilitation outcomes.

## Method

### Search strategy

A search of all studies prior to May 31, 2020 was completed in the following databases: MEDLINE, EMBASE, Cochrane Library, CINAHL, SCOPUS, COMPENDEX, and IEEE Xplore. Keywords were identified using PUBMED MeSH terms of “*mobile applications*”, “*stroke*”, and “*rehabilitation”*. Search strings were created using Boolean operators “OR” and “AND” to combine the keywords. See Additional file 1: Fig. S1 for the sample search strategy used in MEDLINE. Supplementary searching via pearl growing was completed in the included studies.

### Study selection

Studies were included if they were in English and met the following criteria: (1) Randomized control trials (RCTs), quasi-experimental clinical trials, or qualitative studies, (2) study population were adult (18 + years of age) stroke survivors who underwent rehabilitation, and (3) the primary intervention studied was a mobile app (phone, tablet, or PC) on any operating system (e.g., iOS, Android, Windows).

Studies were excluded if they were: (1) reviews, protocols, abstracts, case reports/series, or descriptions of mobile apps, (2) study population were children (< 18 years old) or adults with neurological deficits not secondary to a stroke, or (3) studied mobile apps designed secondarily for another technological tool (e.g., mobile app designed to control robotics devices, functional electrical stimulation, virtual reality headset, telerehabilitation, brain-computer interfaces) or mobile apps part of a larger rehabilitative system requiring additional equipment.

### Screening process

Results from the initial search were imported into Covidence, a systematic review software (Veritas Health Innovation, Melbourne, Australia. Available at www.covidence.org). After duplicates were removed by Covidence, two investigators (S.G.S. and H.W.) independently screened the titles and abstracts through the inclusion and exclusion criteria. The remaining studies were then read in full and assessed for final inclusion eligibility. At the full-text screen phase, reasons for exclusion were recorded and Cohen’s kappa coefficient for inter-rater reliability was calculated. Cohen's kappa coefficient results of ≤ 0 represented poor agreement, 0.01–0.20 was slight agreement, 0.21–0.40 was fair agreement, 0.41– 0.60 was moderate agreement, 0.61–0.80 was substantial agreement, and 0.81–1.00 is almost perfect agreement [21]. At each step, disagreements were discussed between S.G.S. and H.W. before a final decision was made. Prior to screening, it was decided that disagreements that cannot be resolved between S.G.S and H.W would be brought to the remaining authors for a deciding vote. However, this was ultimately not needed.

### Outcomes

Of all the outcomes identified, only those explored in more than 1 study were included in this review. This included three stroke impairments classified as neurological deficits because of a stroke (motor paresis, aphasia, neglect) and five functional outcomes classified as functional improvements that patients make during recovery from a stroke (adherence to exercise, activities of daily living (ADLs), quality of life, secondary stroke prevention, and depression and anxiety).

### Quality evaluation

Risk of bias in the RCTs was assessed using the Cochrane Risk of Bias tool [22]. Bias was divided into low, unclear, or high risk of bias.

The methodological quality of each RCT was analyzed using the modified Downs and Black checklist [23, 24]. RCT methods with a score of 25–27 were considered excellent quality, 19–24 were considered good quality, 14–18 were considered fair quality, and 13 or less were considered poor quality. Since subjects could not be blinded to the intervention, the ‘Intervalidity-Bias’ section had 1 point removed.

### Data extraction

All eligible studies for analysis had data extracted and added to study summary tables. We classified mobile app types into 5 different categories: therapy app, education app, rehab videos, reminders, or a combination. Therapy apps have users interact with the device to complete activities which mimic therapy exercises, often in the form of a game such as finger baseball where users flick their finger on the screen to hit an incoming baseball. Education apps provide a virtual platform for users to learn about stroke and its management. Rehab videos are videos of therapists demonstrating rehabilitation exercises which users can watch and use as a mobile guide to practice with at their leisure. Reminders include messaging texts to remind users to encourage compliance. See Additional file 1: Table S1 for further detailed description of app types. Combinations of mobile apps types that use multiple interventions were also found in these studies. Outcome summary tables were created from RCT extrapolated data, to summarize results based on each outcome and matched to one of the mobile app types.

## Results

### Study selection

**Fig. 1:**
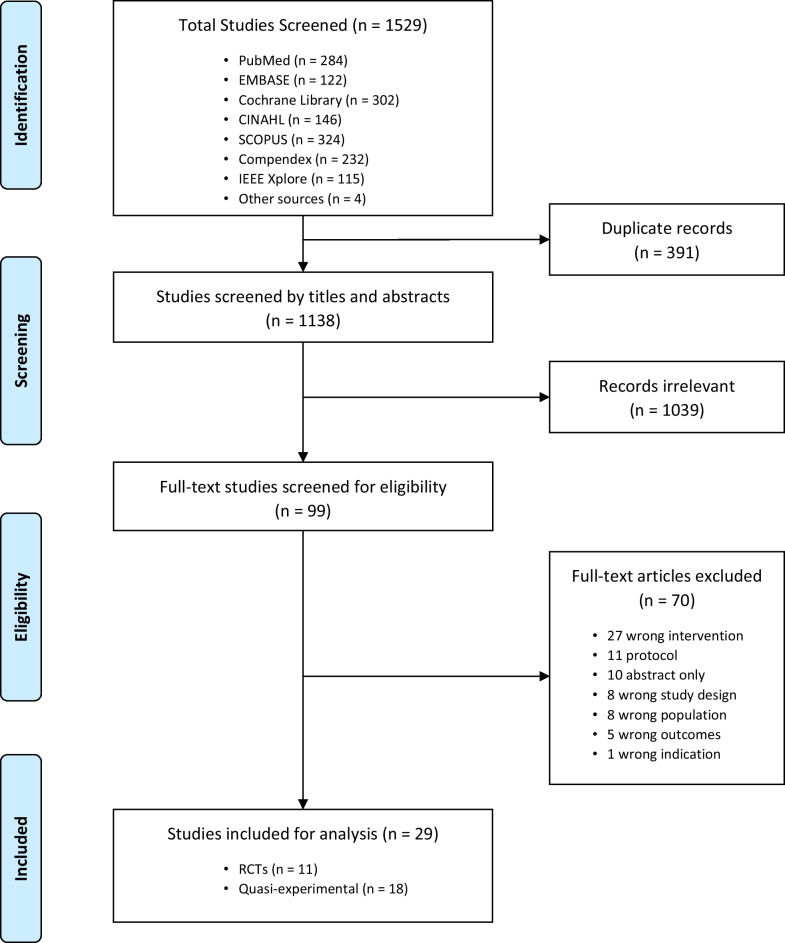
Prisma flow diagram of screening process

The search identified 1529 possible studies for screening, 99 studies underwent a full text review, and ultimately 11 RCTs [16–18, 20, 25–31] and 18 quasi-experimental studies [32–49] met the eligibility criteria for inclusion in this manuscript (Fig. 1). No qualitative study met all the inclusion criteria and thus no qualitative studies were included. A calculated Cohen’s kappa coefficient of 0.75 demonstrated substantial agreement on which studies to include or exclude between reviewers.

### Study quality

Eleven RCTs were assessed for risk of bias (Additional file 1: Fig. S2) and methodological quality (Additional file 1: Table S2). Bias assessment showed that random sequence generation was identified in 7 RCTs, method of allocation concealment was explained in 5 RCTs, blinding of outcome assessments was clear in 7 RCTs, and a low risk of attrition bias was demonstrated in 6 RCTs (Additional file 1: Fig. S3). When comparing methods and pre-published study protocol, only 3 studies were found to have a low risk of reporting bias [17, 20, 30]. In every RCT, participants were not blinded to the intervention group which led to a high risk of performance bias. For methodological quality via the Downs and Black checklist, 4 studies were of excellent quality [18, 25, 28, 31], 6 were of good quality [16, 17, 20, 26, 29, 30], and 1 was of poor quality [27]. Of all the RCTs, 1 study had a high or unknown risk of bias across all domains and poor methodological quality [27]. As quasi-experimental studies have higher inherent levels of bias and poorer methodological qualities than RCTs, no further quality analysis was completed.

### Study characteristics

The characteristics for all 11 RCTs are presented in Table 1. Amongst all RCTs, the study length varied from 0.5 to 12 months with a mode of 1 month. Patient sample size ranged from 16 to 277 with 9 studies having less than 100 patients. The average age for the interventional group was 58.7 ± 11.9 years old and for the control group was 60.1 ± 12.7 years old (Additional file 1: Fig. S4). Between the two groups, the patient ages ranged from 39 to 73 years old. Time since the stroke was between 14 days to approximately 21 months. Three studies did not report the time since stroke [27–29] and 2 studies reported having a control group but did not describe it [16, 27].

**Table 1:** Study characteristics for all 11 RCTs

Author, Year	Length (months)	n	Population Age ± SD	Time since stroke	Intervention	Intervention Description	Comparison
Chung et al., 2020	3	56 IG: 27 CG: 29	Stroke survivors IG: 66.9 ± 14.0 CG: 72.5 ± 15.5	39.5 ± 15.3 days	Rehab videos	Exercise pamphlets with a QR code for exercise videos	Exercise pamphlets
Grau-Pellicer et al., 2020	3	23 IG: 10 CG: 13	Stroke survivors IG: 63.0 ± 11.9 CG: 68.5 ± 11.5	20.85 ± 59.74 months	Therapy app	Fitlab Training and Fitlab Test: accelerometer, GPS, chat messenger	Conventional rehabilitation program
Kamal et al., 2020	12	277 IG: 141 CG: 136	Stroke survivors with a caregiver IG: 60.6 ± 12.0 CG: 59.7 ± 14.3	Unclear	Rehab videos Reminders	Movies4Stroke: videos on exercise, emergencies, meds, secondary prevention plus weekly reminder messages	Conventional rehabilitation program
Kang et al., 2019	1	63 IG: 30 CG: 33	Stroke survivors IG: 50.5 ± 10.8 CG: 52.3 ± 11.0	Unclear	Education app	SHEMA: app containing 12 topics of stroke risk factors	Education booklet with the same 12 topics
Moon et al., 2019	1	16 IG: 8 CG: 8	Stroke survivors with dysphagia IG: 54.13 ± 5.4 CG: 55.4 ± 14.9	22.75 ± 9.21 days	Rehab videos	Instructional videos of orofacial exercises	Exercise booklet guide
Emmerson et al., 2017	1	58 IG: 28 CG: 30	Stroke survivors with upper limb dysfunction IG: 68 ± 15 CG: 63 ± 18	120 (58–226) days	Rehab videos Reminders	HEP videos of the participant performing programs with therapist commentary and daily reminder alarms	Exercises in a written format with diagrams; no reminders
Kang et al., 2017	0.5	21 IG: 10 CG: 11	Stroke survivors with central facial paresis IG: 63.1 ± 10.3 CG: 55.6 ± 16.0	< 12 weeks	Therapy app	Mirror application with reflection and reversal designed for orofacial exercises	Conventional rehabilitation program with SLP therapist
Jang et al., 2016	1	21 IG: 10 CG: 11	Stroke survivors with wrist & finger extensor MRC ≥ 2 IG: 39.3 ± 18.1 CG: 49.3 ± 14.0	963 ± 799 days	Therapy app	Finger training app focused on stretching, flexion, extension, opposition, and thumb abduction	Unclear
Knoche et al., 2016	1	38 IG: 20 CG: 18	Stroke survivors IG:63.6 ± 17.0 CG: 60.7 ± 12.7	Unclear	Therapy app	WAM: moles appear on screen for users to tap	Unclear
Kamal et al., 2015	2	162 IG: 83 CG: 79	Stroke survivors IG: 56.1 ± 1.5 CG: 57.6 ± 1.3	2 (1–5) months	Reminders	SMS reminders to take medication and twice weekly SMS with health information	Conventional rehabilitation program
Prokopenko et al., 2013	0.5	43 IG: 24 CG: 19	Stroke survivors with CI (MCI to mild dementia) IG: 61 [57, 69] CG: 66 [61, 69]	14 days	Therapy app	Computer correction application to improve attention, memory, and visual and spatial gnosis	Conventional rehabilitation program

IG,  intervention group; CG,  control group; QR code,  quick response code; SHEMA,  Stroke Health Education Mobile App; HEP,  home exercise program; SLP,  speech language pathology; WAM,  wack-a-mole; SMS,  short message service

The characteristics for all 18 quasi-experimental studies are presented in Table 2. Thirteen quasi-experimental studies used a pre-test post-test design [32–34, 36, 37, 40–43, 45–49], 3 were a nonrandomized clinical trial [35, 38, 39, 45], and 1 was a crossover design study [43].

**Table 2:** Study characteristics for all 18 quasi-experimental studies

Author, year	Study design	Intervention	Intervention description	Outcome	Result summary
Ballard et al., 2019	Pretest posttest design	Therapy app	Word Trainer: iOS app using ASR to support near-independent practice in word production	Adherence, aphasia scores	No substantial adherence from baseline was shown. However, all participants showed improved word production accuracy and maintenance of gains through the study
Kerry et al., 2019	Pretest posttest design	Therapy app	iReadMore: app aimed to retrain whole-word reading by showing users pictures associated with written and spoken words	Aphasia scores	Using iReadMore increased word-reading accuracy for trained words compared to untrained words
Pugliese et al., 2019	Pretest posttest design	Therapy app	RecoverNow tablet: device preset with specific therapeutic apps for stroke-induced deficits	Adherence	Adherence to intervention was shown to be poor in this study. Barriers to intervention use were categorized as device, patient, and system. App difficulty was the most frequently encountered barrier
Requena et al., 2019	Nonrandomized clinical trial	Reminders	Farmalarm: Android app with visual and audible alerts to increase stroke awareness and treatment adherence	Secondary prevention	The intervention group had more vascular risk factors under control, higher rate of overall vascular risk factors controlled, and higher rate of patients with controlled diabetes mellitus and hypercholesterolemia
Kamwesiga et al., 2018	Nonrandomized clinical trial	Reminders	F@ce intervention: 2 SMS messages per day sent to users to work on three pre-set ADL targets	ADLs	F@ce intervention had no effect on ADLs compared to the control group
Kurland et al., 2018 and 2014	Pretest posttest design (2014 was a pilot test)	Therapy app	HP iBooks: app with series of semantic, phonemic, and orthographic cueing in pictures, words, and videos to help with naming prior pictures	Aphasia scores	Both the 2014 pilot trial and 2017 study suggest that a tablet-based HP programs support maintenance of post-treatment language gains and naming of pictures in individuals with chronic post-stroke aphasia over short and long-term use
Sarfo et al., 2018	Pretest posttest design	Rehab videos	9zest Stroke Rehab App: delivers individualized, goal-targeted 5 days/week video exercise programs	Motor paresis, adherence, ADLs	Higher scores on the Stroke Levity Scale, modified Rankin score, and Barthel’s index score indicates improved motor paresis and less dependence for ADLs. Increased adherence was found in users whose MoCA scores increased with 9zest
Stark et al., 2018	Crossover design	Therapy app	Language Therapy: task based app focused on reading, naming, writing, and comprehension Bejeweled: a spatial awareness and decision mind-game app	Aphasia scores	Aphasia measures via the Comprehensive Aphasia Test and Cookie Theft Picture Description were improved after using the Language Therapy app but not Bejeweled in patients with chronic post-stroke expressive aphasia
Hald et al., 2017	Pretest posttest design	Therapy app	WAM: moles appear on app screen for users to tap with negative or positive feedback	Neglect scores	Negative feedback via WAM resulted in faster reaction times whereas positive feedback led to slower reaction times in stroke patients with hemi-spatial neglect
Lawson et al., 2017	Pretest posttest design	Therapy app	ARMStrokes: app with 8 upper extremity exercises using phone sensors	Motor paresis, ADLs	Increased upper extremity function (PROM, AROM, coordination) and improved ability to perform ADLs
Choi et al., 2016	Pretest posttest design	Therapy app	iAphasia: app with voice-guided tasks based on 6 aphasia therapeutic domains	Aphasia scores	Mean K-WAB scores improved from baseline values, with auditory comprehension, reading, name, and fluency seeing the most benefit
Kizony et al., 2016 Experiment II only	Pretest posttest design	Therapy app	iPad Apps: combined multiple apps (ScribbleKid PegLight, Tap-it, Bowling game) for hand rehab	Motor paresis	Practice effect leading to better performance was more apparent in users with more hand dexterity at baseline
Paul et al., 2016	Nonrandomized clinical trial	Reminders	STARFISH: self and group monitoring app which acts to remind users to do physical activity	Adherence, motor paresis, ADLs, quality of life, secondary prevention, depression/anxiety	Use of STARFISH increased adherence to physical activity but otherwise had no impact on motor function, ADLs, quality of life, secondary prevention, and depression/anxiety
Sureshkumar et al., 2016	Pretest posttest design	Education app	Care for Stroke: delivers information about stroke and post stroke disability management on an app	ADLs	This study showed improvement in ADL markers (Barthel Index and modified Rankin Scale) between before and after the intervention period
Seo et al., 2015	Pretest posttest design	Reminders	KUHMS_2_: app where users record daily values for vascular risk factors, with alarms messages if values were beyond the normal parameters	Secondary prevention	Regarding secondary prevention, the app improved BP and HbA1c values whereas some positive trends were found for waist circumference and smoking cessation
Carabeo et al., 2014	Pretest posttest design	Therapy app	FINDEX: game app focused on fine motor skills via everyday activities	Motor paresis	Fine finger dexterity in the last testing session generally improved compared to the initial measuring session
Hoover et al., 2014	Pretest posttest design	Therapy apps	iPad in ICAP–iPad and a variety of apps were used to enhance therapies (PT, OT, CILT),	Quality of life	SIS and ASHA FACS scores were significantly improved from pre to post treatment period, indicating an increased quality of life through an iPad in ICAP

ADL,  activities of daily living; PROM,  passive range of movement; AROM,  active range of movement; K-WAB,  Korean version of the Western Aphasia Battery; ASR,  automatic speech recognition; WAM,  wack-a-mole; SMS,  short message service; MoCA,  Montreal Cognitive Assessment; HP,  home-practice; KUHMS_2_,  Korea University Health Monitoring System for Stroke; BP,  blood pressure; ICAP,  intensive, comprehensive aphasia program; PT,  physiotherapy; OT,  occupational therapy; CILT,  constraint induced language treatment; SIS,  stroke impact scale; ASHA FACS,  American Speech-Language-hearing Association Functional Assessment of Communication Skills for Adults

### Stroke impairments

Results extrapolated from 7 RCTs studies and 12 quasi-experimental studies examined a range of stroke impairments including motor paresis, aphasia, and neglect.

#### Motor paresis

Six RCTs (Table 3) and 5 quasi-experimental studies (Table 2) explored the effect of mobile apps on various motor paresis metrics (gait/ambulation, standing balance, facial movement, upper extremity or lower extremity range of motion, hand dexterity). Amongst these studies, 4 mobile app types were identified: therapy apps, rehab videos, reminders, and rehab videos with reminders. Therapy apps were used in 3 RCTs [16, 17, 20] and 3 quasi-experimental studies [32, 33, 49]. Each therapy app was designed to promote repetitive motion, often in the form of a game [16, 32, 33, 49]. For therapy apps, all RCTs showed a statistical benefit in a motor paresis metric compared to a control group whereas all quasi-experimental studies demonstrated improvement in a motor paresis metric compared to a pre-test baseline or control group. Rehab videos were used in 2 RCTs [18, 30] and 1 quasi-experimental study [43]. A statistical benefit in upper and lower extremity mobility was found in 1 RCT and 1 quasi-experimental study [18, 43] but 1 RCT [30] showed no statistical improvement of orofacial motor paresis compared to a control group using the standard exercise booklet guide. Reminders to exercise were used in 1 quasi-experimental study [38] and found no positive impact on ambulation (10 m walk test). A single RCT [25] used rehab videos with reminders for 1 month and found no impact on upper extremity function via the Wolf Motor Function Test.

**Table 3:** Results of all the RCTs which explored the effect of mobile app types on a motor paresis metric

	Author, year	Measure	Results	Study conclusion
Therapy apps	Grau-Pellicer et al., 2020	10MWT comfort (m/sec)	IG 1.18 ± 0.35, CG 0.69 ± 0.29 Difference: 0.49 ± 0.06, p = 0.002	Gait speed (10MWT) and walking endurance (6MWT) in the IG improved post-intervention. In the CG, there was a diminishing gait speed and endurance trend For falls risk (TUG), IG improved from fallers to non-fallers. CG remained fallers
		10MWT fast (m/sec)	IG 1.52 ± 0.53, CG 0.85 ± 0.35 Difference: 0.67 ± 0.18, p = 0.002	
		6MWT (m)	IG 380.90 ± 102.69, CG: 238.62 ± 103.81 Difference: 142.28 ± 1.116, p = 0.004	
		TUG (sec)	IG 9.59 ± 3.15, CG 24.42 ± 22.97 Difference: -14.83 ± 19.82 p = 0.057	
	Kang et al., 2017	R-HBGS Mid-face in IG R-HBGS Mid-face in CG	Base: 2.9 ± 0.7, 2-weeks: 2.1 ± 1.0, p < 0.05 Base: 2.5 ± 0.5, 2-weeks: 2.1 ± 0.7, p < 0.05	Compared with the CG, the IG who received orofacial exercises with the use of the tablet PC mirror app showed greater improvement in facial movement after stroke
		R-HBGS mouth in IG R-HBGS mouth in CG	Base: 3.3 ± 1.6, 2-weeks: 2.3 ± 1.6, p < 0.05 Base: 3.5 ± 1.1, 2-weeks: 2.8 ± 1.3, P < 0.05	
		Δ facial movement improvement (mm)	Difference: IG 1.45 ± 0.90, CG 0.55 ± 1, p = 0.04 Ratio: IG: 0.30 ± 0.19, CG: 0.11 ± 0.12, p = 0.01	
	Jang et al., 2016	MMT WE in IG	Base: 3.40 ± 0.84, 4 weeks: 3.80 ± 0.42, p < 0.05	By finger training using the therapy app for 4 weeks, hemiparetic stroke patients achieved functional recovery of the hand and motor recovery of the wrist and hand
		MMT FE in IG	Base: 2.90 ± 0.57, 4 weeks: 3.30 ± 0.67, p < 0.05	
		MFT in IG	Base: 8.10 ± 3.11, 4 weeks: 10.10 ± 3.06, p < 0.05	
		PPT in IG	Base: 3.60 ± 3.37, 4 weeks: 5.20 ± 4.10, p < 0.05	
		MMT (WF, FF) in IG	No statistical difference	
		All MMT, MFT, PPT in CG	No statistical difference	
Rehab videos	Chung et al., 2020	ΔMFAC	IG 1.7 ± 1.2, CG 1.0 ± 1.0, p = 0.036	Video HEP were superior to paper based HEP for mobility gain
	Moon et al., 2019	ΔFDS	IG -11.50 ± 5.32, CG -9.50 ± 4.50, p = 0.368	No significant difference between groups for severity of dysphagia, penetration, or aspiration
		ΔPAS	IG -2.75 ± 0.71, CG -2.63 ± 0.92, p = 0.606	
Rehab videos + reminders	Emmerson et al., 2017	Δ WMFT mean time (sec)	IG -8 ± 13, CG -4 ± 13, p = 0.101	No group differences in upper limb function from HEP videos and reminders vs paper-based HEP
		Δ WMFT grip power (kg)	IG 1.4 ± 2.5, CG 0.9 ± 4.5, p = 0.682	
		ΔWMFT functional score	IG 0.2 ± 0.2, CG 0.2 ± 0.5, p = 0.454	

IG, intervention group; CG, control group; 10MWT comfort, 10 m walk test at a comfortable speed; 10MWT fast, 10 m walk test a maximum speed; 6MWT, 6-min walk test; TUG, timed up and go test; R-HBGS, Regional House-Brackman Grading System; MMT, manual muscle test; WE, wrist extension; WF, wrist flexion; FE, finger extension; FF, finger flexion; PPT, Purdue pegboard test; MFAC, Modified functional ambulatory category; FDS, functional dysphagia scale; PAS, penetration-aspiration scale; HEP, home exercise program; WMFT, Wolf Motor Function test

#### Aphasia

Six quasi-experimental studies (Table 2) used therapy apps to study aphasia, and each showed improvement in aphasia recovery. The mobile app designs focused on expressive and receptive communication by creating visual associations with pictures [40, 45, 46] or using voice recognition software to guide tasks [34, 36, 44]. One study also used a spatial awareness game, Bejeweled, to target chronic (> 1 year) expressive aphasia but it had no impact on recovery [44].

#### Neglect

One RCT (Table 4) and 1 experimental study (Table 2) used a therapy app mimicking a “wack-a-mole” game, where users hit a moving target on the screen, in patients with neglect secondary to a stroke. In the quasi-experimental study [37], the wack-a-mole game also included positive and negative auditory feedback in the form of a ring. The game started with a base ring and each successful “hit” of a mole resulted in a higher pitched sound (positive feedback). When a mole was missed, the pitch restarted back to the base ring (negative feedback). The RCT [27] (with no auditory feedback) had no significant effect on any measure of neglect. The quasi-experimental study found that an added auditory feedback significantly improved reaction time but did not correlate reaction time to neglect outcomes.

**Table 4:** Results of all the RCTs which explored the effect of mobile app types on neglect

	Author, year	Measure	Results	Study conclusion
Therapy apps	Knoche et al., 2016	SDMT	No significant effects	No significant effects of WAM play time on any measure of neglect
		CBS observer	No significant effects	
		CBS insight deficit	No significant effects	

IG, intervention groupCG; control group; SDMT, symbol digit modality test; CBS, Catherine Bergego scale; WAM, wack-a-mole

### Functional outcomes

Results extrapolated from 8 RCTs studies and 10 quasi-experimental studies examined a range of functional outcomes including adherence to exercise, ADLs, quality of life, secondary stroke prevention, and depression and anxiety.

#### Adherence to exercise

Three RCTs (Table 5) and 4 quasi-experimental studies (Table 2) assessed adherence to exercise using therapy apps, rehab videos, reminders, or a combination of rehab videos with reminders. One RCT [20] and 2 quasi-experimental studies [36, 47] used therapy apps and measured exercise adherence. The RCT [20] showed an improvement in adherence to ambulation and the 2 quasi-experimental studies [36, 47] found that therapy apps did not improve adherence to exercise. Rehab videos were used in 1 RCT [18] and 1 quasi-experimental study [43] to measure exercise adherence. The RCT by Chung et al. found that rehab videos significantly improved exercise adherence after 3 months but not after 1 month. The quasi-experimental study [43] using rehab videos demonstrated a correlation between improved exercise adherence and higher scores on the Montreal Cognitive Assessment test. Reminders used in 1 quasi-experimental study [38] found an improvement in exercise adherence. In 1 RCT [25], a combination of rehab videos with reminders was found to have no impact on exercise adherence.

**Table 5:** Results of all the RCTs which explored the effect of mobile app types on adherence to exercise

	Author, year	Measure	Results	Study conclusion
Therapy apps	Grau-Pellicer et al., 2020	Ambulation (min/d)	IG_A_ 90.85 ± 83.88, CG 34.00 ± 31.07, p = 0.034	Statistically significant increases in adherence to ambulation and reduction of sitting time found in the IG compared to the CG
		Sitting time (hours/day)	IG_A_ 4.40 ± 2.22, CG 9.84 ± 5.89, p = 0.012	
Rehab videos	Chung et al., 2020	Adherence VAS	Base: IG 74.1 ± 24.4, CG 64.1 ± 34.0, p = 0.214 1-month: IG 73.7 ± 21.5, CG 58.6 ± 37.3, p = 0.072 3-months: IG 75.6 ± 26.2, CG 55.2 ± 35.8, p = 0.021	Mobile video-guided HEP was superior to standard paper-based HEP in terms of exercise adherence for patients recovering from stroke
Rehab videos + reminders	Emmerson et al., 2017	% of HEP done/day	IG 62 ± 25, CG 60 ± 28, p = 0.785	In stroke survivors with upper limb impairment, no group differences in exercise adherence found between the IG and CG
		Min/day doing HEP	IG 34 ± 20, CG 43 ± 38, p = 0.293	
		Hours with OT	IG 8.3 ± 6.2, CG 8.0 ± 5.8, p = 0.871	

IG, intervention group; CG, control group; VAS, visual analog scale; MMAS, Morisky medication adherence scale; HEP, home exercise program; OT, occupational therapy

#### Activities of daily living (ADLs)

Five RCTs (Table 6) and 5 quasi-experimental studies (Table 2) tracked ADL independence after patients used a therapy app, education app, rehab video, reminders, or rehab videos with reminders. Three RCTs [20, 26, 27] and 1 quasi-experimental study [32] used a therapy app. Of the RCTs, one study [20] showed a statistically significant benefit in ADLs as per the Barthel Index whereas the other 2 did not [26, 27]. The 1 quasi-experimental study [32] found its therapy app improved performing ADLs. An education app providing information on stroke and post-stroke management was used in 1 quasi-experimental study [42] and showed improvement in ADLs. Rehab videos used in 1 RCT [18] showed no benefit in ADLs while 1 quasi-experimental study [43] found rehab videos improved ADL independence. Reminders used in 2 quasi-experimental studies [38, 39] had no effect on ADLs. One RCT [28] used a combination of rehab videos with reminders and found no significant benefit in ADL independence after 1 year.

**Table 6:** Results of all the RCTs which explored the effect of mobile app types on activities of daily living

	Author, year	Measure	Results	Study conclusion
Therapy apps	Grau-Pellicer et al., 2020	BI	IG 97.50 ± 5.40, CG 84.62 ± 14.21 Difference: 12.88 ± 8.81, p = 0.013	Post-intervention, the IG improved from mildly dependent to independent for ADLs whereas the CG remained mildly dependent for ADLs
	Knoche et al., 2016	FIM Motor	No data reported	No significant effects of WAM play time on FIM motor or FIM cognitive scores
		FIM Cognitive	No data reported	
	Prokopenko et al., 2013	iADL scale	IG: 20.5 [13, 24] 20.5 [18, 24], p = 0.1 CG: 17 [13, 20] 16 [14.5,21], p = 0.123	No significant changes found in the IADLs, possibly due to the short study period and small sampling
Rehab videos	Chung et al., 2020	Δ mBI	IG 20.9 ± 13.9, CG 19.4 ± 13.1, p = 0.808	Exercise videos were not superior to paper-based exercise programs for basic ADL gain for patients
Rehab videos + reminders	Kamal et al., 2020	% treatment arm BI: 0–50	Base: IG 50.3%, CG 50.3%, p = 0.94 6-months: IG 16.3%, CG 17.8%, p = 0.16 1-year: IG 14.0%, CG 18.3%, p = 0.35	Baseline IG and CG had equal % of participants with total to severe dependency for ADLs. At 6 and 12 months, a smaller percentage of the IG had total to severe dependency compared to the CG

IG, intervention group; CG, control group; ADLs, activities of daily living; BI, Barthel Index; mBI, modified Barthel Index; FIM, Functional Independence Measure

#### Quality of life

Three RCTs (Additional file 1: Table S3) and 2 quasi-experimental studies (Table 2) examined the impact on quality of life after using either a therapy app, education app, or reminders. The effect of different therapy apps on quality of life was studied in 2 RCTs [20, 26] and 1 quasi-experimental study [41]. One RCT [20] and the quasi-experimental study [41] found therapy apps improved patient perceived quality of life whereas the other RCT [26] did not. One RCT [29] using an education app with information on stroke risk factors found no significant benefit in quality of life. One quasi-experimental study [38] used reminders through a self and group monitoring app and found it had no impact on quality of life.

#### Secondary stroke prevention

Three RCTs (Additional file 1: Table S4) and 3 quasi-experimental studies (Table 2) used education apps, reminders, or rehab videos with reminders to measure impact on secondary stroke prevention. One RCT [29] used an education app to teach users about stroke risk factors. The post-study questionnaire results showed a non-significant increase in stroke risk factor knowledge. One RCT [31] and 3 quasi-experimental studies [35, 38, 48] used reminders to improve vascular risk factors. One RCT [31] and 1 quasi-experimental study [38] found that reminders made no significant impact on blood pressure. However, the other 2 quasi-experimental studies [35, 48] showed significant improvement in controlling several vascular risk factors such as blood pressure, glycemic control, lipid levels, and BMI. One RCT used rehab videos (Movies4Stroke) with reminders and had no significant change in control of hypertension, LDL, or HbA1.

#### Depression and anxiety

Depression and anxiety were studied in 1 RCT (Additional file 1: Table S5) using a therapy app and 1 quasi-experimental study (Table 2) using reminders. The RCT’s therapy app used therapy-like games with corrective help features to train cognition [26]. Neither the RCT [26] using its therapy app nor the quasi-experimental study [38] using reminders had a benefit on depression and anxiety.

## Discussion

The purpose of this systematic review was to explore mobile apps for stroke rehabilitation and the stroke impairments and functional outcomes for which they have shown to be effective. Specifically, we delved into the impact of varying mobile app types on stroke rehabilitation. There was wide variation in the effectiveness of apps across several studies. This perhaps is not surprising given the variability in apps and stroke impairments described in the different studies.

Technology, as a method of delivery (for therapy, education, or reminders), should mirror what is found to be effective in non-technological methods of delivery. For example, in education there have been numerous systematic reviews and meta-analysis showing that online provision of education results in similar outcomes to face-to-face teaching [50–52]. However, the concepts which do improve outcomes (interactivity, feedback, repetition, practice exercises, social learning) are what makes learning effective in either online and face-to-face delivery of education [53]. Similarly, it is also important to consider beneficial concepts of effective face-to-face stroke rehabilitation therapy (massed practice, task-specific practice, goal-oriented practice, multisensory stimulation, rhythmic cueing, feedback, social interaction, and constraint-induced therapy) when evaluating mobile applications [54–56]. However, we need to be mindful of the benefits and challenges of technology enhanced delivery methods to find the right approach for the right outcome. There is also mounting evidence, including recent systematic reviews and meta-analyses, that integrating online and face-to-face delivery methods together (known as blended learning) produces improved outcomes over either alone [53, 54]. Although there is a paucity of evidence to determine if face-to-face therapy combined with mobile apps is better than either intervention alone, this would be an interesting area for future research.

Mobile apps, in combination with face-to-face delivery of stroke rehabilitation, may afford us with benefits such as augmenting therapy (types and time) for stroke deficits, providing stroke education, delivering rehab videos, and sending reminders, but further research is needed into how we can best use them to support stroke recovery.

Through this review, 3 stroke impairments were identified that may benefit from app usage: motor paresis (upper and lower extremity dexterity and coordination, gait training, orofacial paresis), aphasia, and neglect.

After a stroke, more than 70% of people will suffer from motor impairments including upper or lower extremity paresis [57]. Given this high prevalence, it is not surprising that motor paresis was the most studied deficit in this review. Eight of these studies conferred a positive impact on motor paresis by mobile apps during stroke rehabilitation, including 4 RCTs [16–18, 20]. Of the 2 RCTs [25, 30] that showed no impact, the study by Moon et al. had the smallest sample size (n = 16) amongst all RCTs and no power analysis, raising concerns that it may be underpowered. As well, it focused on post-stroke dysphagia, which may be harder to facilitate through videos than more obvious limb motions. There were 4 types of mobile apps that were studied (therapy apps, rehab videos, reminders, and a combination of rehab videos with reminders), with therapy apps being the most studied type. The main principle of each therapy app was to guide repetitive movement in the affected extremity, sometimes in the form of games [33, 49]. This aligns with face-to-face research that shows repetition of meaningful movements, such as with ADLs, resulted in better functional outcomes [58, 59]. Apps may also allow serious games and gamification principles to motivate patients to persist with exercise repetitions [60]. Overall, there is strong evidence indicating that therapy apps targeted for various motor paresis metrics are highly effective in stroke rehabilitation. Rehab videos also had potential as 1 RCT [18] and 1 quasi-experimental study [43] showed benefit. As for the use of reminders with or without rehab videos, there were only 2 studies [25, 38] which explored these types, none of which conferred any motor benefits.

Aphasia is also a common consequence of stroke, affecting 35% of patients [61]. In a 2020 systematic review and meta-analysis, there was evidence supporting the use of face-to-face constraint-induced aphasia therapy which focuses on forcing patients to use verbal language with massed practice [62]. In this review, 6 quasi-experimental studies [34, 36, 40, 44–46] explored the impact on aphasia using mobile apps built on similar principles to constraint-induced aphasia. In all the studies, aphasia scores were found to improve after each study period. All 6 studies used a therapy type of app. Most commonly, the therapy apps used repetitive language exercises in combination with a stimulus (pictures, written words, voice-guided tasks) to develop expressive and receptive communication skills. Only one study used a task based therapy app and Bejeweled, a spatial awareness and decision making game, to rehabilitate chronic aphasia [44]. In this study, the task-based therapy app improved domains of aphasia whereas the game Bejeweled did not. This is suggestive that therapy apps which focus on repetitive communication through various language exercises may assist in the rehabilitation of post-stroke aphasia.

For neglect, the evidence for stroke rehabilitation apps is mixed, similar to the literature on the impact of face-to-face visual scanning on post-stroke neglect [63, 64]. In this review, a single RCT [27] showed its therapy app had no impact on neglect but the study methodology scored poorly on the Downs and Black checklist and there were multiple bias concerns as per the Cochrane Risk of Bias tool. In 2017, the same authors published a quasi-experimental study exploring the same app but with a feedback system and this led to improved user reaction time, however no measure of neglect was used [37]. The improvement in reaction time may be due to dual channel assumption (using both auditory and visual channels to encode information) which is one aspect of the cognitive theory of multimedia learning and has been proven to be superior to using only one channel [65–67]. This may have further implications and should be considered in future research in stroke apps. Lastly, the small screen size of apps may have a negative outcome with respect to neglect or visuoperceptual therapy.

Within this review, 5 functional outcomes were identified after a review of the literature: adherence to exercise, ADLs, quality of life, secondary stroke prevention, and depression and anxiety. For each of these outcomes, there was mixed evidence for effectiveness.

Of these outcomes, adherence to exercise had the most positive impact from mobile apps use, with 2 out of 3 RCTs and 2 out of 4 quasi-experimental studies showing improvement. The 2 RCTs [18, 20] that showed an increase in exercise adherence had a study length of 3 months and an average time since stroke of less than 40 days. In comparison, the RCT [25] that showed mobile apps had no impact on exercise adherence had a short study length (1 month) and longer average time since stroke (120 days). Assuming increased exercise adherence leads to increased therapy time, mobile apps that promote adherence may have the potential to improve functional gain [10] since early rehabilitation results in better recovery up to 6 months since onset [68, 69]. Therefore, apps that promote exercise adherence earlier in stroke recovery and for longer duration may be more beneficial for increasing exercise time and possibly stroke outcomes, but further research is needed.

Exercise adherence showed the most improvement with rehab videos, having 1 RCT [18] and 1 quasi-experimental study [43] in support of this and no studies against. This again may point to the importance of dual channel learning and the impact that videos may have through the ability to revisit information and utilizing educational principles such as spaced repetition and distributed practice [65, 70]. Other than rehab videos, the remaining app types (therapy apps, reminders, reminders with rehab videos) used to study exercise adherence showed limited or mixed results. In two of the studies demonstrating increased adherence [20, 38], the mobile apps allowed for user interactions via a chat messenger or group monitoring, so that fellow stroke users could encourage one another. This built a social support network, which has known benefits on improving therapy adherence [71].

For ADLs, quality of life, secondary prevention control, and depression and anxiety this review found that the effect of mobile apps is inconclusive, regardless of the type. This suggests that further research is required in these specific areas.

A summary of the effects of mobile applications for each stroke impairment and functional outcome along with the relevant principles of effective education and face-to-face stroke rehab therapy are placed into Table 7.

**Table 7:** Summary table of the results and the principles of effective face-to-face interventions (education and stroke rehab therapy) used in the beneficial mobile app types for each stroke impairment and functional outcome

		Summary	Principles of effective education	Principles of effective face-to-face stroke rehab therapy
Stroke impairments	Motor paresis	11 studies explored motor paresis metrics after use of a therapy app, rehab videos, reminders, or a combination of rehab videos with reminders. Only 8 studies (4 RCTs) demonstrated an improvement in motor paresis. Of the mobile app types, therapy apps had the most significant positive impact. The therapy apps were designed for users to focus on repetitive motor movements through interactive activities, often as games	Repetition Interactivity Practice exercises	Massed practice Task-specific practice Goal-oriented practice Constraint-induced therapy Social interaction
	Aphasia	6 studies (no RCTs) explored aphasia recovery after use of a therapy app and all the studies showed an improvement in aphasia recovery. The therapy apps had users practice expressive and receptive communication by completing tasks with visual cues and auditory prompts	Interactivity Practice exercises	Task-specific practice Goal-oriented practice Rhythmic cueing Multisensory stimulation Constraint-induced therapy
	Neglect	2 studies explored the impact on neglect after use of a therapy app and 0 studies showed a significant benefit	N/A	N/A
Functional outcomes	Adherence to exercise	7 studies explored adherence to exercise after use of a therapy app, rehab videos, reminders, or a combination of rehab videos with reminders. Only 4 studies (2 RCTs) showed an improvement in adherence to exercise, with rehab videos of repeated exercises having the most significant and consistent impact. The other app types showed limited or mixed results. 2 studies that showed a positive adherence to exercise included a feedback feature from other users	Repetition Feedback Practice exercises Social learning	Social interaction Massed practice Task-specific practice Goal-oriented practice Feedback
	Activities of daily living	10 studies explored activities of daily living after use of therapy app, education app, rehab video, reminders, or rehab videos with reminders. The results were mixed as only 4 studies (1 RCT) demonstrated benefit. The 1 RCT that showed significance had a chat messenger with other users for group interaction and feedback	Interactivity Feedback Social learning	Social interaction Feedback
	Quality of life	5 studies explored quality of life after use of therapy apps, education app, or reminders. The results were mixed as only 2 studies (1 RCT) demonstrated benefit. The 1 RCT that showed significance had a chat messenger with other users for group interaction and feedback	Interactivity Feedback Social learning	Social interaction Feedback
	Secondary stroke prevention	7 studies explored secondary stroke prevention after education apps, reminders, or rehab videos with reminders. Only 2 studies (0 RCTs) demonstrated a significant impact on select measured outcomes	N/A	N/A
	Depression and anxiety	2 studies explored the impact on depression and anxiety after use of a therapy app or reminders. 0 studies showed a significant benefit	N/A	N/A

There are several limitations within this review. First is the lack of high-quality studies in the body of literature on mobile apps for stroke rehabilitation as 18 of the eligible 29 studies were quasi-experimental studies, which carry a high risk of bias due to its methodology. For this reason, the most important recommendation for improving study quality is to have randomization to reduce bias in all other aspects of the study. As well, blinding where possible is important as blinding patients is challenging with technology, hence leading to a high risk of patient bias. One potential method to overcome the challenge of blinding patients with technology is to create a ‘control’ mobile application that does not directly relate to the outcome. For example, for a study exploring the effects of a stroke rehab mobile application on motor paresis, a ‘control’ mobile application could be designed to provide education on stroke prevention. Additionally, amongst the 11 RCTs examined in this review, there were several limitations noted in addition to those previously mentioned. Most of the RCTs generally had small sample sizes and only 4 trials [20, 25, 29, 31] showed a power analysis. This raises concerns of underpowered studies and thus, minimizes its clinical implications. The study follow-up times are also important to consider. Study lengths varied from 0.5 to 12 months with a mode of 1 month. One of the challenges of conducting clinical trials in the early and late sub-acute periods is that an intervention during this time period has the potential to have longer lasting impacts on stroke recovery out to 6 months or even longer [72, 73]. Consideration needs to be given to longer follow-up periods for these studies, even if the intervention itself is briefer (e.g., 4–6 weeks). Across the literature, many in-person interventions require several weeks to months to lead to a positive change [74–76]. The dose and length of time an intervention needs to be administered may be dependent on the specific problem being treated. Future research would benefit from having higher quality studies by using a ‘control’ mobile app, randomization, having larger sample sizes, and longer follow-up periods. For future mobile applications for stroke rehabilitation, we suggest incorporating features that we already have evidence for in face-to-face education and stroke rehabilitation therapy such as inter-user interactions to develop social feedback and encouragement, practice exercises with recommended number of structured repetitions, task-specific and goal-oriented practices, constraint-induced therapy, and direct user-interactivity with the mobile application. Of note, we recognize that several national agencies are looking to regulate “software as a medical device” and apps will fall under these regulations. However, at this time, we would not recommend a regulatory body for standardization given the infancy of this field and that regulations can limit the creativity of mobile application development for stroke rehab.

As the demand for limited healthcare resources continues to rise due to multiple factors such as the COVID19 pandemic and aging population [77–79], technology has developed a larger role in patient care. COVID-19 has also necessitated increased technology usage leading to improved comfort level by clinicians, patients and caregivers [80, 81]. However, the integration of new technology continues to be limited due to multiple challenges such as clinical acceptance, user learning curve, privacy and security, and the lack of funding models that support the use of technology to augment therapy [82–86]. Future studies should also examine real world use of mobile apps to examine barriers of implementation such as mobile app feasibility and privacy, organizational resource and time use, and motivating factors for patients and/or healthcare providers use [87]. Despite these barriers, mobile applications continue to be an area of growing interest in stroke rehabilitation based on the rising numbers of new studies. With the advantages described in this review and the rapid evolution and acceptance of technology, mobile applications for stroke rehabilitation appear to be a potentially exciting field for research expansion.

## Conclusions

This systematic review provides evidence that mobile apps can be used to improve stroke rehabilitation, particularly in combination with face-to-face therapy for motor paresis, aphasia, and adherence to exercise. When mapping out app types, there were several studies in support of using therapy apps for motor paresis and aphasia, rehab videos for exercise adherence, and reminders for exercise adherence. Since stroke rehabilitation inpatients spend much of their time sedentary, providing cost effective mobile apps for therapy may bring patients closer to, or even exceed, the standards set by the Canadian Stroke Best Practices and American Heart Association/American Stroke Association [8, 9, 11–13], potentially improving stroke recovery. With the ubiquitous presence of smartphones, there has been a growing accessibility to devices and comfort with technology utilization which also paves a path for increased uptake [88]. Technology acceptance has also been accelerated with necessitated use since the COVID-19 pandemic [80]. Although changing the way we provide therapy may be met with resistance and challenges, it is imperative that we continue to strive to provide the best evidence-based stroke rehabilitation possible, examining the advantages, disadvantages and opportunities associated with technology enhanced therapy provision. With this potential, there is a need for further research to better understand the impact of mobile apps on varying types on stroke deficits and functional outcomes, both alone and in combination with face-to-face stroke rehabilitation. Future studies would benefit in having higher quality RCTs with less reporting and attrition bias (especially for aphasia), larger sample sizes with power analysis, increased study duration of at least 6 months, studies focused on mobile applications with characteristics of face-to-face therapy, clustering patient populations to stoke lesion and acuity, and usability studies to improve user experiences.

## Supplementary Information

**Additional file 1. **Additional materials, additional Figures S1–S4, additional Tables S1–S5.

## Data Availability

Not applicable.

## Abbreviations

CAD: Canadian dollar
USD: United States dollar
SD: Standard deviation
RCT: Randomized control trial
ADLs: Activities of daily living
IG: Intervention group
CG: Control group
QR code: Quick response code
SHEMA: Stroke health education mobile app
HEP: Home exercise program
PT: Physiotherapy
OT: Occupational therapy
SLP: Speech language pathology
WAM: Wack-a-mole
SMS: Short message service
PROM: Passive range of movement
AROM: Active range of movement
K-WAB: Korean version of the Western Aphasia Battery
ASR: Automatic speech recognition
MoCA: Montreal cognitive assessment
HP: Home-practice
KUHMS_2_: Korea University Health Monitoring System for Stroke
BP: Blood pressure
LDL: Low-density lipoproteins
ICAP: Intensive, comprehensive aphasia program
CILT: Constraint induced language treatment
SIS: Stroke impact scale
ASHA FACS: American Speech-Language-hearing Association Functional Assessment of Communication Skills for Adults
10MWT comfort: 10 M walk test at a comfortable speed
10MWT fast: 10 M walk test a maximum speed
6MWT: 6 Minute walk test
TUG: Timed up and go test
R-HBGS: Regional house-Brackman grading system
MMT: Manual muscle test
WE: Wrist extension
WF: Wrist flexion
FE: Finger extension
FF: Finger flexion
PPT: Purdue pegboard test
MFAC: Modified functional ambulatory category
FDS: Functional dysphagia scale
PAS: Penetration-aspiration scale
WMFT: Wolf motor function test
SDMT: Symbol digit modality test
CBS: Catherine Bergego scale
VAS: Visual analog scale
MMAS: Morisky medication adherence scale
BI: Barthel Index
mBI: Modified Barthel index
FIM: Functional independence measure
SS-QOL-2: Stroke specific quality of life scale
EQ-5D-5L: EuroQoL 5-dimension 5-level instrument
HADS: Hospital anxiety and depression scale
