# Supplementary material for: Effect of mobile application types on stroke rehabilitation: a systematic review
Source: J Neuroeng Rehabil. 2023 Jan 24;20:12. doi: 10.1186/s12984-023-01124-9 (PMC9872745; doi:10.1186/s12984-023-01124-9)
Supplement: Supplementary file 1 — Additional file 1. Additional materials, additional Figures S1–S4, additional Tables S1–S5. [file 12984_2023_1124_MOESM1_ESM.docx]

**Additional file 1**

**Additional materials**

1. Mobile applications
   - ((“mobile applications” [MeSH] OR "mobile application*") OR ("computers, handheld" [MeSH] OR "handheld computers" OR "handheld computer*") OR ("cell phone" [MeSH] OR “cell phone*”) OR (“smartphone” [MeSH] OR “smartphone*”) OR (app OR apps OR mHealth OR mHealth* OR iPhone* OR iPad* OR Android OR Android* OR tablet*))
2. Stroke
   - (“stroke” [MeSH] OR stroke OR stroke* OR strokes* OR “cerebrovascular accident*” OR "cerebrovascular event*" OR CVA)
3. Rehabilitation
   - ((“rehabilitation” [MeSH] OR rehab OR rehab*) OR (“physical and rehabilitation medicine” [MeSH] OR "physical and rehabilitation medicine" OR physiatry OR physiatrist*) OR (“physical therapy specialty” [MeSH] OR “physical therapy modalities” [MeSH] OR physiotherapy OR physio*) OR (“occupational therapy” [MeSH] OR “occupational therp*”) OR (“rehabilitation of speech and language disorders” [MeSH] OR “speech language patholog*” OR “speech language therap*” OR speech* OR language OR language*) OR (“exercise” [MeSH] OR exercise OR exercise*))

**Figure S1.** Search string used in MEDLINE combined 1 AND 2 AND 3.

**
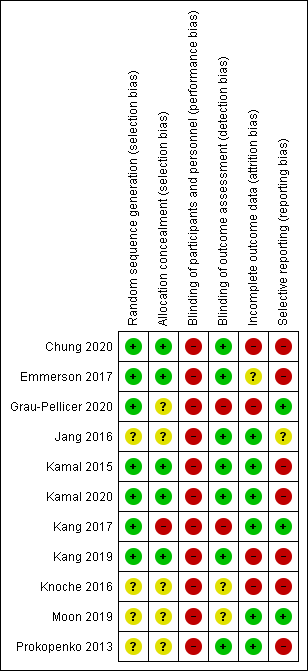
**

**Figure S2.** Risk of bias summary for the individual RCTs. (+) represents low risk of bias, (?) represents unclear risk of bias, and (–) represents high risk of bias.


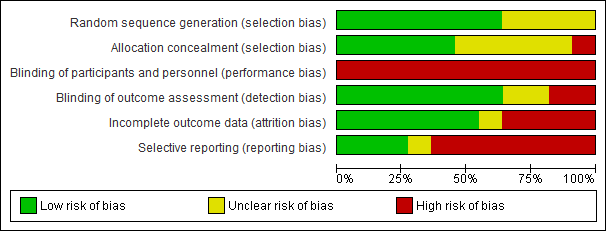


**Figure S3.** Risk of bias graph for RCTs by percentage of risk.


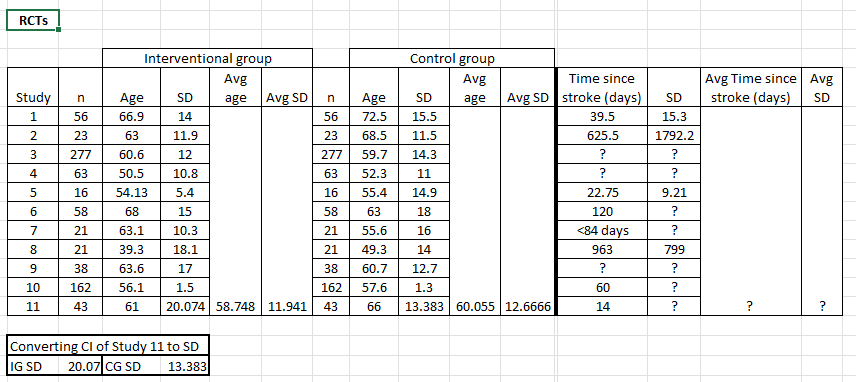


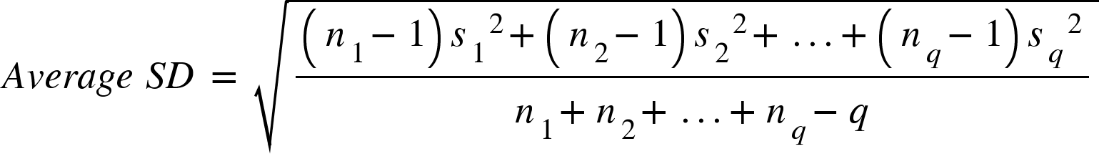


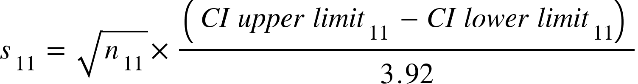


- n_q_ is the sample size for Study “q”
- s_q_ is the standard deviation for Study “q”
- q is the total number of studies
- CI upper limit_11_ is the upper limit of the confidence interval from Study 11
- CI lower limit_11_ is the lower limit of the confidence interval from Study 11

**Figure S4.** Excel sheet with RCT raw data, calculations, and relevant equations to identify the average age and associated SD for the ‘interventional groups’ and ‘control groups’. Due to insufficient data for ‘Time since stroke’, the same calculations could not be completed.

| **App type** | **Description** | **Example from a study** |
| --- | --- | --- |
| Therapy app | Mobile app with an interactive activity (often as a game) to reproduce therapy sessions one would participate with PT, OT, or SLP | **Jang et al., 2016** (16)  Interactive touch screen games for users to work on hand and finger dexterity (stretching, flexion, extension, opposition, and thumb abduction) |
| Education app | Mobile app which provides text and pictures to explain basic pathophysiology of a stroke, expected signs and symptoms, diagnostic methods, and management guidelines | **Sureshkumar et al., 2016** (42)  Mobile app with information about stroke and post stroke disability management through text and pictures |
| Rehab videos | Mobile app with a video that shows users how to practice therapy while on their own time | **Moon et al., 2019** (30)  This app has instructional videos of orofacial exercises which the user can follow along and practice |
| Reminders | Mobile app with an alarm that reminds users to perform various tasks such as to exercise or to take medication | **Kamal et al., 2015** (31)  SMS reminders to uses to take medication and twice weekly SMS with health information |
| Combination | Mobile app which uses a combination of one of the above app types | **Emmerson et al., 2017** (25)  Rehab videos + reminders: Home exercise program videos of the participant performing therapy exercises and daily reminder alarms |

**Table S1.** Characterization of the 5 app types, including a description and a study example.

| **Study** | **Reporting (11)** | **External validity (3)** | **Internal validity: bias (6*)** | **Internal validity: confounding (6)** | **Power (1)** | **Total (27*)** |
| --- | --- | --- | --- | --- | --- | --- |
| **Chung et al., 2020** | 11 | 3 | 6 | 5 | 0 | 25 |
| **Grau-Pellicer et al., 2020** | 11 | 3 | 4 | 4 | 1 | 23 |
| **Kamal et al., 2020** | 10 | 3 | 6 | 6 | 0 | 25 |
| **Kang et al., 2019** | 10 | 2 | 5 | 5 | 1 | 23 |
| **Moon et al., 2019** | 10 | 1 | 5 | 3 | 0 | 19 |
| **Emmerson et al., 2017** | 11 | 3 | 6 | 5 | 1 | 26 |
| **Kang et al., 2017** | 10 | 2 | 5 | 3 | 0 | 20 |
| **Jang et al., 2016** | 10 | 3 | 6 | 5 | 0 | 24 |
| **Knoche et al., 2016** | 5 | 2 | 3 | 3 | 0 | 13 |
| **Kamal et al., 2015** | 10 | 3 | 5 | 6 | 1 | 25 |
| **Prokopenko et al., 2013** | 10 | 1 | 6 | 4 | 0 | 21 |

**Table S2.** Downs and Black methodological analysis of RCTs. IG is intervention group and CG is control group. Parenthesis numbers in the first row represents total score for the respective category. Grading: 25-27 (excellent quality), 19-24 (good quality), 14-18 (fair quality), and ≤13 (poor quality). *Cumulative score for ‘Internal Validity – Bias’ and thus Total Score was dropped by one as no study could blind subjects given the nature of the intervention.

|  | Author, Year | Measure | Results | Study Conclusion |
| --- | --- | --- | --- | --- |
| Therapy Apps | Grau-Pellicer et al., 2020 | EQ-5D-5L | IG_A_ 8 ± 1.82, CG 12.54 ± 3.71, p = 0.002 | At baseline, IG and CG perceived limitations that mildly-moderately affected quality of life. After the intervention, IG perceived limitations mildly affecting quality of life whereas CG had no change. |
|  | Prokopenko et al., 2013 | SS-QOL-2 | IG: 156.5 [134,180] 🡪 155.5 [134,180], p = 0.1  CG: 153.5 [142,163] 🡪 155.5 [134,180], p = 0.2 | No significant changes found in quality of life, possibly due to a short study period and small sampling. |
| Education Apps |  |  |  |  |
|  | Kang et al., 2019 | EQ-5D-5L | IG 0.62 ± 0.29, CG 0.46 ± 0.41, p = 0.07 | Both groups had a non-significant increase in quality of life. Change in EQ-5D-5L was marginally greater in IG compared to CG |

**Table S3.** Results of all the RCTs which explored the effect of mobile app types on quality of life. IG = intervention group. CG = control group. SS-QOL-2 = Stroke Specific quality of life scale. EQ-5D-5L = EuroQoL 5-dimension 5-level instrument.

|  | Author, Year | Measure | Results | Study Conclusion |
| --- | --- | --- | --- | --- |
| Education Apps | Kang et al., 2019 | Stroke-knowledge questionnaire | Post-test comparison  IG 29.07 ± 5.27, CG 28.00 ± 5.46, p = 0.43 | Knowledge of stroke risk factors improved in each group but the difference between groups was not statistically significant. |
|  |  |  |  |  |
| Reminders | Kamal et al., 2015 | Δ sBP in IG vs CG | 1.0 mmHg, p = 0.678 | Trend towards reduced diastolic blood pressure in IG vs. CG |
|  |  | Δ dBP in IG vs CG | 2.6 (-5.5 to 0.15) mmHg, p = 0.06 |  |
|  |  |  |  |  |
| Rehab Videos + Reminders | Kamal et al., 2020 | % treatment arm sBP<125 mmHg | Base: IG 50.0, CG 50.0, p = 0.96  6-months: IG 50.0, CG 50.0, p = 0.61  12-months: IG 62.0, CG 38.0, p = 0.16 | Movies4Stroke failed to achieve its primary outcome variable of the control of hypertension, LDL cholesterol, or HbA_1c_ in IG compared the CG. Post-intervention, there were greater percentages of participants with sBP<125, dBP<85, and A_1c_<7in the IG vs the CG. However, none of these results were statistically significant. |

**Table S4.** Results of all the RCTs which explored the effect of mobile app types on secondary stroke prevention. IG = intervention group. CG = control group. s/dBP = systolic/diastolic blood pressure. LDL = low-density lipoproteins.

|  | Author, Year | Measure | Results | Study Conclusion |
| --- | --- | --- | --- | --- |
| Therapy Apps | Prokopenko et al., 2013 | HADS - *Anxiety* | Base: IG 6 [4,8], CG 8 [5,9], p = 0.2  2-weeks: IG 6 [5,8], CG 8 [5,10], p = 0.2 | Degree of anxiety and depression after completing the therapy has not changed significantly in both groups. |
|  |  | HADS - *Depression* | Base: IG 7 [3,7], CG 8 [5,10], p = 0.07  2-weeks: IG 3 [2,8], CG 6.5 [4.5,8.5], p = 0.2 |  |

**Table S5.** Results of all the RCTs which explored the effect of mobile app types on depression and anxiety. IG = intervention group. CG = control group. HADS = hospital anxiety and depression scale.
